# Cases of Syphilis

**Published:** 1826-11

**Authors:** 

**Affiliations:** Surgeon to the Middlesex Infirmary.


### SYPHILIS.

### *Cases of Syphilis, treated with Scruple Doses of Calomel,* by Mr. Boyle, Surgeon to the Middlesex Infirmary.

I.—Henry Pitt, ætatis eighteen, a young man of delicate and slight habit, came under my care as a patient of the Middlesex Infirmary, on the 17th June, 1826, for the treatment of a venereal chancre situated behind the corona glandis superiorly, and a small but painful bubo in the right groin. Had been taking mercurial pills for four days, but the mouth was not affected.

Gave him a scruple of Calomel, with one grain of Opium, at bedtime.

18th.—Mouth sore; chancre evidently improved in appearance; bubo stationary. The bowels had been moved twice in the morning, without griping or uneasiness.

19th.—Chancre improving in appearance; state of the mouth and bubo stationary.—No medicine.

20th.—Chancre healed; mouth more tender; the bubo, however, notwithstanding the early and continued use of the ordinary repellent measures, is larger: the fluctuation of matter is distinct, and the skin in the centre has become thin, is peeling off, and appears about to break.

Moxa to be applied round the base of the enlarged glands, followed by the application of adhesive straps over the part.

21st.—Bubo evidently diminished in size; state of the mouth the same as it was yesterday.

Blue-pill and Antimonial Powder, of each two grains, night and morning.—The moxa and straps to be continued.

22d.—Bubo much reduced, the matter being all absorbed, leaving a depression in the centre, marking the situation previously occupied by it.—The moxa, straps, and blue-pill, were continued till the 28th, when the patient was discharged cured.

II.—C. C. consulted me on the 26th July, 1826, respecting a sore, about half the size of a sixpence, situated outside of the prepuce, on the left side of the penis. His statement was, that a few days after connexion with a suspicious person, he observed a small pustule containing matter, and, apprehending that it was syphilitic, he applied caustic, which was productive of the sore described. From this account, and the appearances which the sore then exhibited, I hesitated to administer mercury, and advised a trial of local measures. Accordingly, the black wash, the red precipitate ointment, and other applications, were employed; and the patient lived temperately, and took alterative medicine: the sore, however, remained stationary as to size, and acquired a hard cartilaginous margin. It was now (after a week's trial of the above means) irritable, and bled on being touched. Considering it imprudent any longer to withhold the use of mercury, I ordered a scruple of Calomel, with a grain and a half of Opium, to be taken going to bed. On the following morning, the mouth was slightly affected, the bowels had been twice opened, and the sore was cleaner. No medicine was then ordered; but on the following evening, the mouth being nothing more affected, the dose was repeated.

On the next day, the mouth was more affected, and the sore, which was covered by a healthy-looking matter, exhibited, on being washed, numerous granulating points. The bowels on this occasion were not once moved.

Four days after the second dose, the sore was healed; but, the action of the scruple doses having been then somewhat lessened, the patient was ordered to take Blue-pill and Antimonial Powder, of each three grains, for a few days after.—This gentleman had been treated in the manner above stated for a similar affection in 1822.

III.—Mr. —— came to town for advice on the 23d of August. He had a well-marked chancre, of the Hunterian character, situated between the frenum and glans on the left side of the penis. This gentleman, whilst in the country, had tried caustic and various other applications to no purpose, for a period of a fortnight. The sore was still of moderate size, had a hardened base, and bled on being touched.

A scruple of Calomel, with a grain and a half of Opium, going to bed. No dressing except dry lint.

24th.—Had one copious evacuation without griping. Mouth slightly affected; chancre cleaner, red, and healthy looking.

25th.—Mouth not quite so tender; sore improving.

The Calomel and Opium, to the same extent, to be repeated going to bed.

27th.—Mouth more affected; sore cicatrising.

28th.—Chancre skinned over; state of the mouth stationary.

To have two grains of Blue-pill, and an equal quantity of Antimonial Powder, night and morning.

On the 30th, the mouth becoming more affected than was deemed essential, the medicine was altogether left off. In this case, as well as in the preceding one, the bowels were not once moved by the second dose, but continued regular throughout the treatment.

It will be manifest that the exhibition of the blue-pill and antimonial powder, practised in the foregoing cases, was to keep up the action excited by the scruple doses of calomel, for a reasonable time after the healing of the sores, and thus avoid the possibility of producing unnecessary effects by the introduction of another large dose of calomel. This modification in my practice of treating syphilis by large doses of the submuriate of mercury, as laid down in my treatise on that subject, I have followed from the time of its publication; and the results have been uniformly satisfactory.

*4, Cleveland-square, St. James's; Sept. 2d,* 1826.

